# Author Correction: Evidence for oxygen-conserving diamond formation in redox-buffered subducted oceanic crust sampled as eclogite

**DOI:** 10.1038/s41467-026-69139-z

**Published:** 2026-02-02

**Authors:** Sonja Aulbach, Thomas Stachel

**Affiliations:** 1 Institut für Geowissenschaften, Goethe-Universität, Frankfurt am Main, Germany; 2 Earth and Atmospheric Sciences, University of Alberta, Edmonton, AB Canada

**Keywords:** Mineralogy, Petrology

Correction to: *Nature Communications* 10.1038/s41467-022-29567-z, published online 8 April 2022

Since publication of the article, it has come to the authors’ attention that the first author’s spreadsheet used for the iterative pressure-temperature estimates for eclogites and pyroxenites contained an error, necessitating corrections to the text, Tables 1 and 2, Figs. 1, 2 and 4, Supplementary Fig. 1 and Dataset 1.

The correction shifted temperature-pressure estimates to significantly higher average values, including to values >1500 °C, which are not considered here in keeping with other work^1^ as it may reflect disequilibrium, leading to a change in the number of samples with temperature-pressure estimates. The corresponding shift in the absolute ∆logƒO_2_(FMQ) estimates resulting from the new pressure-temperature estimates is modest, from a mean ∆logƒO_2_(FMQ) of –3.31 to a mean ∆logƒO_2_(FMQ) of –3.50. In addition, the number of samples, coefficients and *p*-values for some correlations changed slightly.

We further detected an error when double-checking against the second author’s spreadsheet for the iterative pressure-temperature estimates for eclogites and pyroxenites via geotherm projections^2^. A corrected version is posted at 10.5683/SP3/IMYNCL.

We noted that *f*O_2_ values for five samples from the northern Slave craton were shown in the figures but missing from Supplementary Table 1. This is now corrected.

The Supplementary information accompanying this amendment lists the changes made to the text and shows the original, uncorrected Tables 1 and 2 and Figs. 1, 2 and 4 for comparison. The corrections have been made to the HTML and PDF versions of the article.

The first author would like to apologise for any inconvenience caused. We thank Dr. Giulia Marras for uncovering the error.

## Supplementary information

Explanation of changes
