# Supplementary material for: Evidence for oxygen-conserving diamond formation in redox-buffered subducted oceanic crust sampled as eclogite
Source: Nat Commun. 2022 Apr 8;13:1924. doi: 10.1038/s41467-022-29567-z (PMC8993838; doi:10.1038/s41467-022-29567-z)
Supplement: Supplementary file 1 — Supplementary Information [file 41467_2022_29567_MOESM1_ESM.pdf]

Evidence for oxygen-conserving diamond formation in redox-buffered subducted oceanic crust (eclogite)

Sonja Aulbach[1,*], Thomas Stachel[2]
[1] Institut für Geowissenschaften, Goethe-Universität, Frankfurt am Main, Germany
[2] Earth and Atmospheric Sciences, University of Alberta, Edmonton, AB, Canada

*Corresponding author. *E-mail address*: s.aulbach@em.uni-frankfurt.de

Supplementary Information

Supplementary Table 1 Parental melt composition modelling based on Wang et al. (2019)[33]

Δ log *f*O_2	FMQ-2	FMQ-1	FMQ	FMQ+1
~Warm Archaean MORB: *F* = 0.2 and T_P = 1450 °C (1549 °C at *F* = 0.2)				
Peridotite-melt Bulk *D(V)*	0.23	0.14	0.09	0.05
V in aggregated melt (ppm)	**174**	239	301	345
~Cool Archaean MORB: F = 0.2 and T_P = 1400 °C (1499 °C at *F* = 0.2)				
Peridotite-melt Bulk *D(V)*	0.26	0.16	0.10	0.06
V in aggregated melt (ppm)	154	217	282	333
~Modern MORB: F = 0.08 and T_P = 1300 °C (1347 °C at *F* = 0.08)				
Peridotite-melt Bulk *D(V)*	0.73	0.44	0.27	0.16
V in aggregated melt (ppm)	81	131	**206**	314

fO_2 oxygen fugacity; FMQ Fayalite-Magnetite-Quartz; *F* melt fraction; T_P mantle potential temperature; *D* distribution coefficient

Note: The spreadsheet of Wang et al. (2019)[33] calculates V distribution coefficients for spinel peridotite as a function of temperature and *f*O_2 (and of mineral compositions which were adopted from the spreadsheet) and melt compositions at 1 GPa, whereby temperature increases with increasing *F*; temperature for the first melt increment is taken to be T_P + 0.4*30, using the adiabat of Katsura et al. (2010)[34] and assuming 30 km = 1 GPa where the melt last equilibrates with its source; V abundances are for aggregated fractional melts

V concentrations in the melt for suggested Archaean and modern conditions are highlighted in bold

Supplementary Table 2 Cumulate composition modelling for sequential mineral crystallisation

Sample	*D(V)* min-melt	Wt. fraction	Min V ppm	Bulk V ppm
~Warm Archaean MORB with parental V concentration = 170 ppm at T_{XX} = 1340 °C and fO_2 = FMQ-2				
Spinel	6.94	0.01	1180	13
Olivine	0.15	0.20	26	5
Plagioclase	0.06	0.28	10	3
Bulk sp-ol-pl cumulate	0.12	0.49		**21**
Clinopyroxene	2.9	0.16	493	79
BULK+cpx				100
BULK+primitive melt		0.47		100
~Warm Archaean MORB with parental V concentration = 300 ppm at T_{XX} = 1340 °C and fO_2 = FMQ				
Spinel	2.95	0.01	885	10
Olivine	0.05	0.20	15	3
Plagioclase	0.02	0.28	6	2
Bulk sp-ol-pl cumulate	0.05			**14**
Clinopyroxene	0.85	0.34	255	85
BULK+cpx				100
BULK+primitive melt		0.29	300	100
~Cool Archaean MORB with parental V concentration = 154 ppm at T_{XX} = 1290 °C and fO_2 = FMQ-2				
Spinel	7.76	0.01	1194	13
Olivine	0.16	0.20	25	5
Plagioclase	0.07	0.28	10	3
Bulk sp-ol-pl cumulate	0.14			**21**
Clinopyroxene	3.77	0.14	581	79
BULK+cpx				100
BULK+primitive melt		0.52	154	100
~Cool Archaean MORB with parental V concentration = 154 ppm at T_{XX} = 1290 °C and fO_2 = FMQ				
Spinel	3.29	0.01	507	6
Olivine	0.05	0.20	8	2
Plagioclase	0.02	0.28	3	1
Bulk sp-ol-pl cumulate	0.05			**8**
Clinopyroxene	0.71	0.84	109	92
BULK+cpx				100
BULK+primitive melt		0.33	154	100

fO_2 oxygen fugacity; *FMQ* Fayalite-Magnetite-Quartz buffer; *F* melt fraction; T_{XX} crystallisation temperature converted from T_P mantle potential temperature using the relationship given in Herzberg and Asimow (2008)[35]; *D* distribution coefficient; min. mineral; wt. weight; sp spinel, ol olivine, pl plagioclase, cpx clinopyroxene

Parental melt V concentrations for modelled conditions and initial melt fraction of 0.2 taken from Supplementary Table 1; V concentrations in bulk cumulates of spinel+olivine+plagioclase shown in bold font; weight fraction of clinopyroxene and primitive melt were adjusted such that the resultant mixture with bulk cumulate yields 100 ppm, corresponding to the low end of abundances in gabbroic eclogites (Fig. 3)

Note: Mineral-melt distribution coefficients for spinel peridotite as a function of temperature and fO_2 are from the spreadsheet of Wang et al. (2019)[33], which also considers mineral compositions (adopted from the spreadsheet); V abundances are for sequential crystallisation of spinel, olivine, plagioclase and clinopyroxene at weight fractions that were modelled for fractional crystallisation of picrite at 0.05 GPa by Aulbach and Jacob (2016, ref. [36]; their Appendix 5), ignoring, for simplicity, small differences in phase relations arising from differences in fO_2 (example in ref. [36]), and decreasing temperatures with progressive crystallisation

Supplementary Table 3 Melt composition modelling for fractional crystallisation

	Melt F	Melt MgO (wt.%)	Temperature-fO_2 conditions				
T_P			1450 °C	1450 °C	1450 °C	1500 °C	1400 °C
Parent melt V			170	240	300	190	100
T_{XX}			1340 °C	1340 °C	1340 °C	1380 °C	1290 °C
Δ log fO_2			FMQ-2	FMQ-1	FMQ	FMQ-2	FMQ-2
			D(V) mineral-melt				
Spinel			6.94	4.52	2.95	6.37	0.76
Olivine			0.15	0.07	0.05	0.14	0.16
Plagioclase			0.06	0.03	0.02	0.06	0.06
			Remaining melt V (ppm)				
Onset ol XX	0.99	16.8	159	231	294	179	100
Onset pl XX	0.79	9.8	194	286	366	218	122
Onset cpx XX	0.51	8.4	363	548	704	410	228

fO_2 oxygen fugacity; FMQ Fayalite-Magnetite-Quartz buffer; F melt weight fraction remaining; T_{XX} crystallisation temperature converted from T_P mantle potential temperature using the relationship given in Herzberg and Asimow (2008, ref. [35]); D distribution coefficient; ol olivine, pl plagioclase, cpx clinopyroxene

Parental melt V concentrations for modelled conditions taken from Supplementary Table 1 and mineral-melt distribution coefficients from Supplementary Table 2 or modelled as described therein and in the Methods

Note: V abundances are for sequential fractionation of spinel, olivine, plagioclase and clinopyroxene, and for weight fractions of the remaining melt, ignoring, for simplicity, small differences in phase relations arising from differences in fO_2 (example in Aulbach and Jacob, 2016, ref. [36]), and decreasing temperatures with progressive crystallisation; melt weight fractions as well as melt MgO content from ref. [36] (their Appendix 5) which were modelled for fractional crystallisation of picrite at 0.05 GPa

Supplementary Table 4 Eclogite composition modelling for high-pressure batch melt extraction

Element ppm (Δ log fO_2)	IW	V (FMQ-4)	V (FMQ-2)	V (FMQ-1)	V (FMQ)	Ce/Yb_{NMORB}
C_0		170	170	170	170	
D rutile-melt		4.96	3.89	3.36	2.82	
D cpx-melt	5.4	6.49	2.14	1.14	0.57	
D garnet-melt	4.2	6.49	2.14	1.14	0.57	
Bulk *D* eclogite-melt		6.48	2.15	1.15	0.58	
		Concentration (ppm) and ratio in residue from batch melting				
Melt *F* = 0.05		177	174	171	164	0.65
Melt *F* = 0.1		185	179	172	158	0.47
Melt *F* = 0.2		204	190	174	149	0.29
Melt *F* = 0.3		227	202	176	140	0.19

fO_2 oxygen fugacity; IW Iron-Wuestite buffer, FMQ Fayalite-Magnetite-Quartz buffer; *F* melt fraction; *D* distribution coefficient; cpx clinopyroxene

C_0 for V is chosen as an example, for Ce and Yb it corresponds to concentrations in NMORB of Gale et al. (2013, ref. [31]); *D*(V) rutile-melt and *D*(V) cpx-melt as a function of fO_2 were parameterised from results reported in Holycross and Cottrell (2020, ref. [37]) and Mallmann and O'Neill (2009), respectively; D(V) garnet-melt was assumed to be identical to that of cpx based on results reported in Mallmann and O'Neill (2009, ref. [38]); cpx-melt *D*(Ce) and *D*(Yb) from Barth et al. (2002, ref. [39]); bulk D was calculated for 0.005 rutile, 0.445 cpx and 0.55 garnet following Aulbach and Jacob (2016, ref. [36]); Ce/Yb_{NMORB} is the NMORB-normalised ratio

Average of mineral-melt *D*(V) for experiments carried out near the iron-wuestite (IW) oxygen buffer are from Barth et al. (2002, ref. [39])

Supplementary Table 5 Metasomatic clinopyroxene modelling

	Temperature-fO_2 conditions					
Metasomatic melt V (ppm)	100	150	100	150	100	150
T_{XX}	1100 °C	1100 °C	1200 °C	1200 °C	1200 °C	1200 °C
Δ log fO_2	FMQ	FMQ	FMQ	FMQ	FMQ+1	FMQ+1
	D(V) clinopyroxene-melt					
	3.53	3.53	1.84	1.84	1.00	1.00
	V concentration (ppm) in clinopyroxene					
	353	530	184	276	100	150

fO_2 oxygen fugacity; FMQ Fayalite-Magnetite-Quartz buffer; T_{XX} crystallisation temperature; *D* distribution coefficient; cpx clinopyroxene

Metasomatic clinopyroxene is assumed to crystallise from a kimberlite-like melt with V concentrations of 100-150 ppm, reflecting average concentrations in different kimberlites from the Superior craton as examples (Tappe et al., 2017, ref. [40]); cpx-melt distribution coefficients as a function of temperature and fO_2 derived from Wang et al. (2019, ref. [33]) as described in Supplementary Table 2 and in the Methods

Supplementary Table 6 Ti and V concentrations in synthetic rutile and in three samples determined by EPMA compared to LAM-ICPMS

Sample	TiO_2	Total V	Apparent V	% Total V	Corrected V	LAM-ICPMS V	% Deviation EPMA
	wt.%	ppm	ppm[1]		ppm	ppm[2]	vs LAM
Avg dl cpx	0.03	152					
Avg dl gt	0.03	159					
Synth Rutile (13)	99.73	2660	2660				
1σ	0.45	68	68				
OE23 cpx (6)	0.49	279	13	5	266	252	5
1σ	0.01	42	0	1	42		
OE16 cpx (2)	0.36	445	10	2	436	455	-4
1σ	0.01	11	0	0	11		
OE34 cpx (9)	0.35	216	9	5	207	198	4
1σ	0.01	59	0	2	59		
OE23 gt (6)	1.01	219	27	13	192	188	3
1σ	0.02	45	0	3	45		
OE16 gt (3)	0.19	118	5	9	113	101	12
1σ	0.01	90	0	11	90		

EPMA Electron Probe Micro Analyser, LAM-ICPMS Laser Ablation Microprobe-Inductively-Coupled Plasma Mass Spetrometer; cpx clinopyroxene, gt garnet, dl detection limit, synth synthetic; number in parentheses = spots measured

[1] V concentration arising from the overlap of TiKβ on the VKα peak, as determined by measurement of V_2O_3 in the pure synthetic rutile standard and amounting to 26.7±0.7 ppm V per wt.% TiO_2

[2] V concentration reported in Aulbach et al. 2020 (ref. [14])

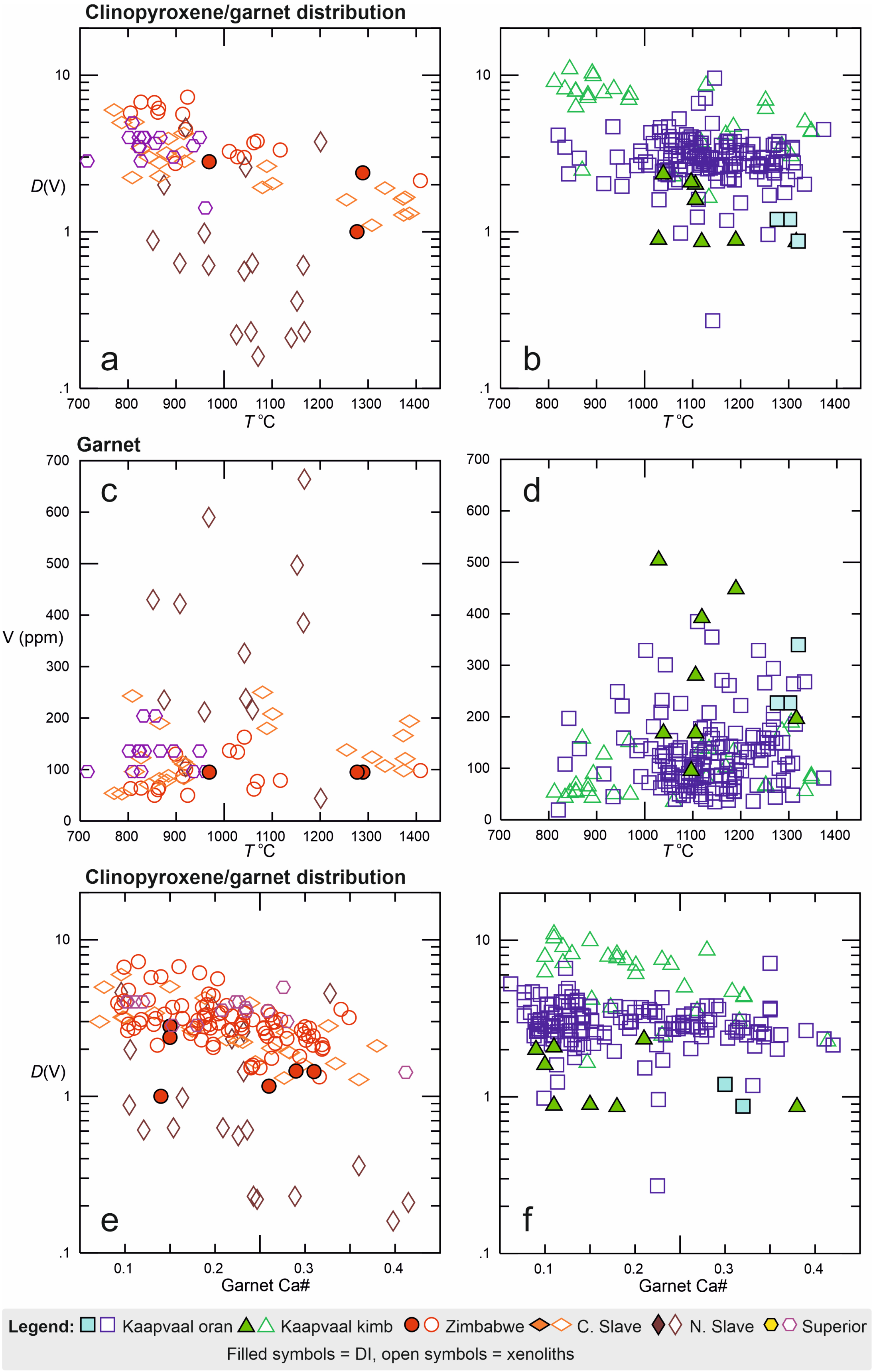
Clinopyroxene/garnet distribution
D(V)
T °C
a
b
Garnet
V (ppm)
c
d
Clinopyroxene/garnet distribution
Garnet Ca#
e
f
Legend: Kaapvaal oran Kaapvaal kimb Zimbabwe C. Slave N. Slave Superior
Filled symbols = DI, open symbols = xenoliths

28
29

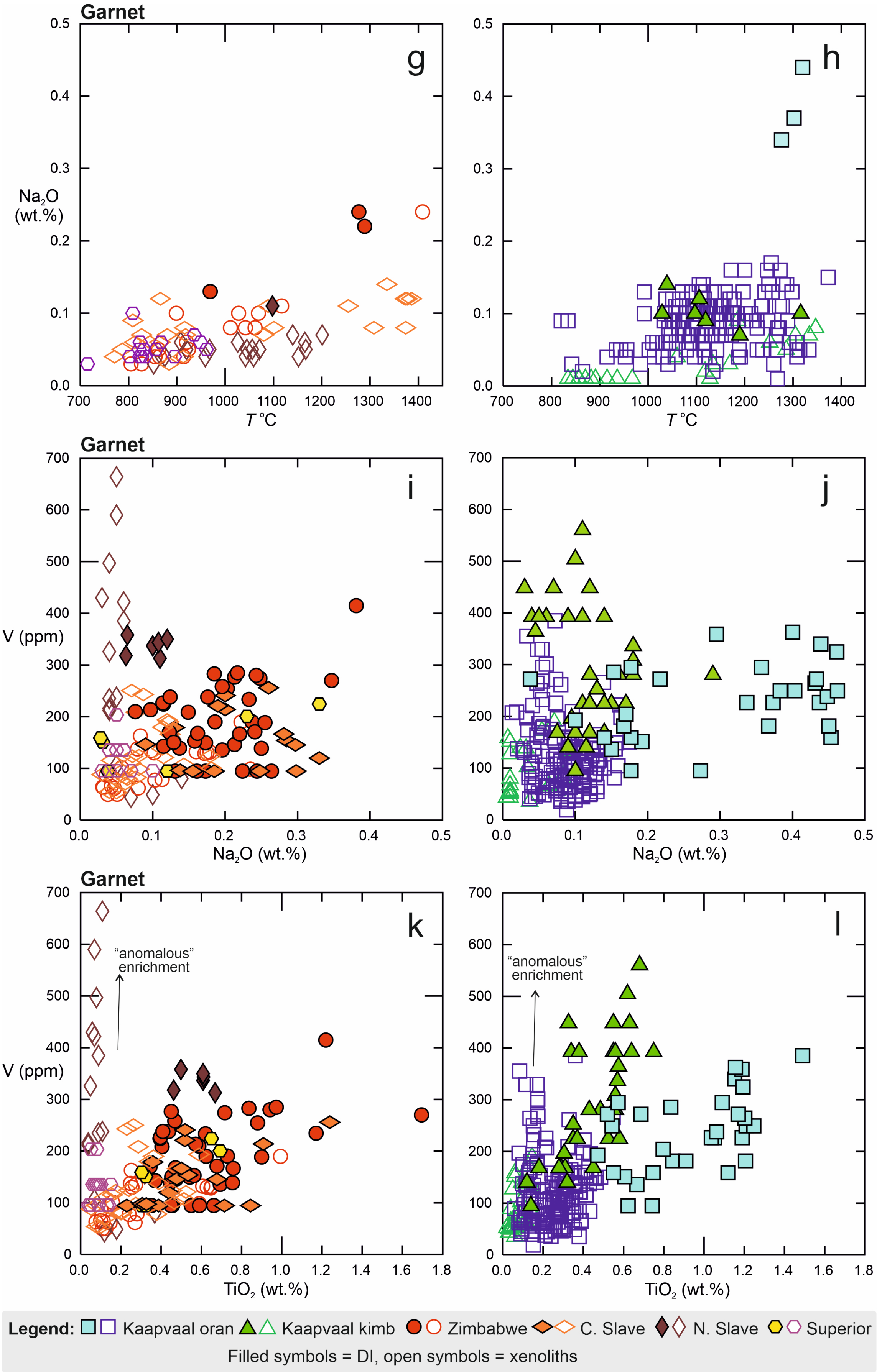
Garnet
g
h
Na2O (wt.%)
T °C
Garnet
i
j
V (ppm)
Na2O (wt.%)
Garnet
k
l
"anomalous" enrichment
V (ppm)
TiO2 (wt.%)
Legend: Kaapvaal oran Kaapvaal kimb Zimbabwe C. Slave N. Slave Superior
Filled symbols = DI, open symbols = xenoliths

30
31

Supplementary Fig. 1 Scatter plots illustrating relationships between various variables suggested to be related to temperature-crystal-chemically-controlled uptake of V and other minor and trace elements. **a-b** Distribution *D* of V between clinopyroxene and garnet in eclogite xenoliths and DI as a function of temperature (°C; thermometer of Krogh, 1988, ref. [41], calculated iteratively with regional conductive model geotherms; see Methods); **c-d** V contents (ppm) in garnet as a function of temperature (°C); **e-f** *D*(V) as a function of Ca# (Ca/(Mg+Fe^{total}+Ca+Mn) molar); **g-h** Na_2O contents (wt.%) in garnet as a function of temperature (°C); **i-j** V abundances in garnet (ppm) as a function of Na_2O contents (wt.%); **k-l** V abundances in garnet (ppm) as a function of TiO_2 content (wt.%). Several samples from Kaapvaal and the northern Slave craton show anomalous (relative to the main trend) enrichment in V; data for different localities are split up into two panels to avoid clutter, those for the Kaapvaal craton show orangeite-hosted (oran) and kimberlite-hosted (kimb) samples, reflecting two distinct periods of magmatism, separately. Average 1σ uncertainties on V abundances in garnet from DI are 117 ppm and from xenoliths 5.6 ppm (corresponding to typical uncertainties for multiple analyses per sample reported in the literature, see Methods). Data sources in Supplementary Data 1.

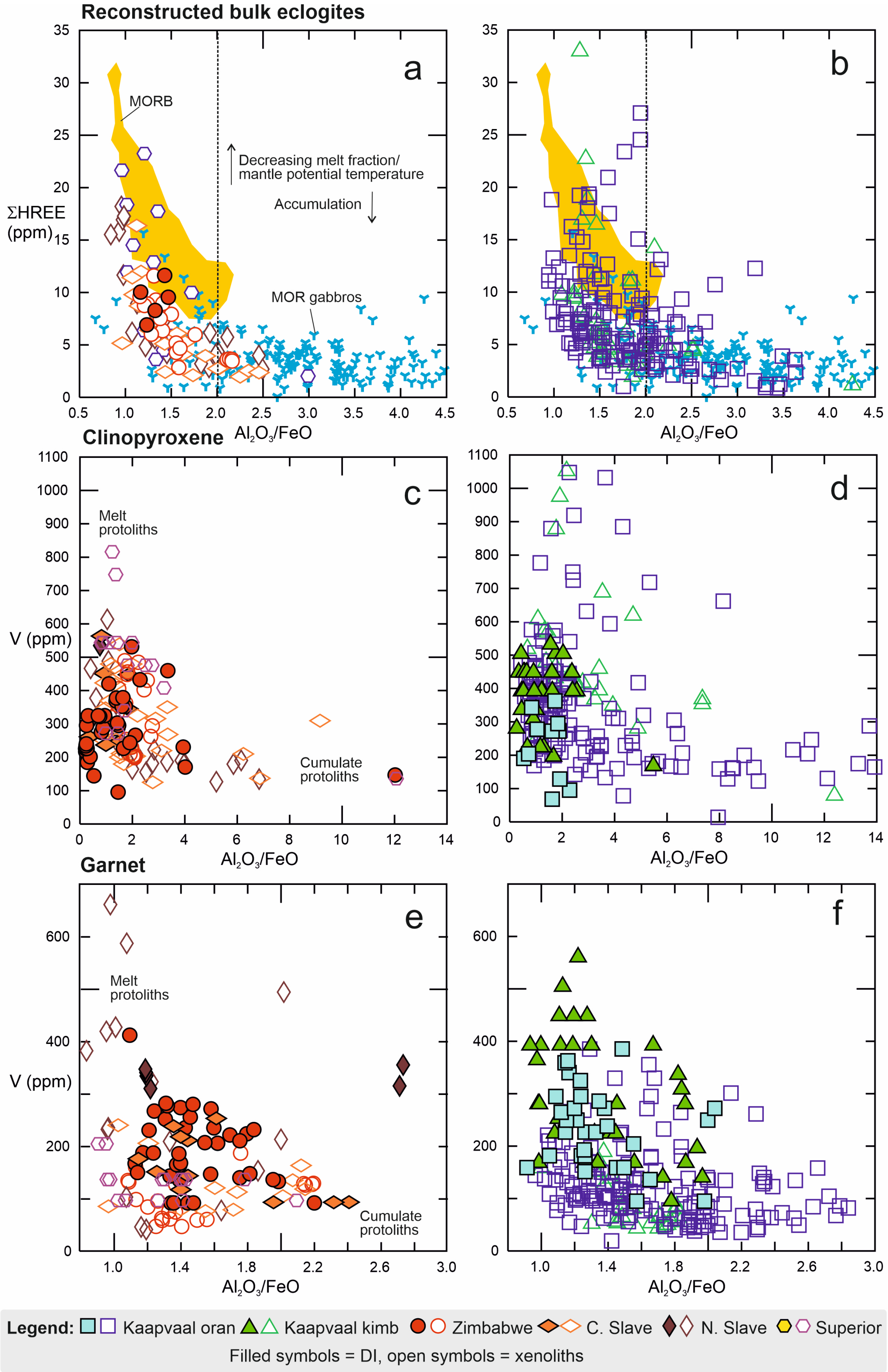
Reconstructed bulk eclogites
a
b
MORB
ΣHREE (ppm)
Decreasing melt fraction/ mantle potential temperature
Accumulation
MOR gabbros
Al2O3/FeO
Clinopyroxene
c
d
Melt protoliths
V (ppm)
Cumulate protoliths
Al2O3/FeO
Garnet
e
f
Melt protoliths
V (ppm)
Cumulate protoliths
Al2O3/FeO
Legend: Kaapvaal oran Kaapvaal kimb Zimbabwe C. Slave N. Slave Superior
Filled symbols = DI, open symbols = xenoliths

Supplementary Fig. 2 Scatter plots illustrating the effect of accumulation and differentiation, using Al_2O_3/FeO as a proxy for plagioclase accumulation during low-pressure protolith formation, on trace element and V abundances in eclogite. **a-b** ΣHREE (ppm; summed from Tb to Lu) in reconstructed eclogite xenoliths and DI. Effect of accumulation, and of decreasing mantle potential temperatures and lower resulting melt fractions, are indicated with arrows in **a**, also shown for comparison are mid-ocean ridge basalts (MORB; yellow field; data from Jenner and O'Neill, 2012, ref. [42]) and MOR gabbros (blue tristars, Eu/Eu*>1.05; from PetDB: www.earthchem.org/petdb). Stippled line shows suggested cut-off between cumulates and melts; there are few diamonds with clinopyroxene-garnet pairs from which bulk rocks can be reconstructed, and few of those with REE data. Vanadium abundances (ppm) in **c-d** clinopyroxene and **e-f** garnet. Samples with suggested cumulate vs. melt protoliths are indicated. Average 1σ uncertainties on V abundances in clinopyroxene, garnet and reconstructed bulk eclogites from DI are 67, 117 and 69 ppm, respectively, from xenoliths they are 14.5, 5.6 and 29 ppm, respectively (corresponding to typical uncertainties for multiple analyses per mineral and sample reported in the literature, and to propagated uncertainties for reconstructed bulk eclogites, see Methods). Data sources in Supplementary Data 1.

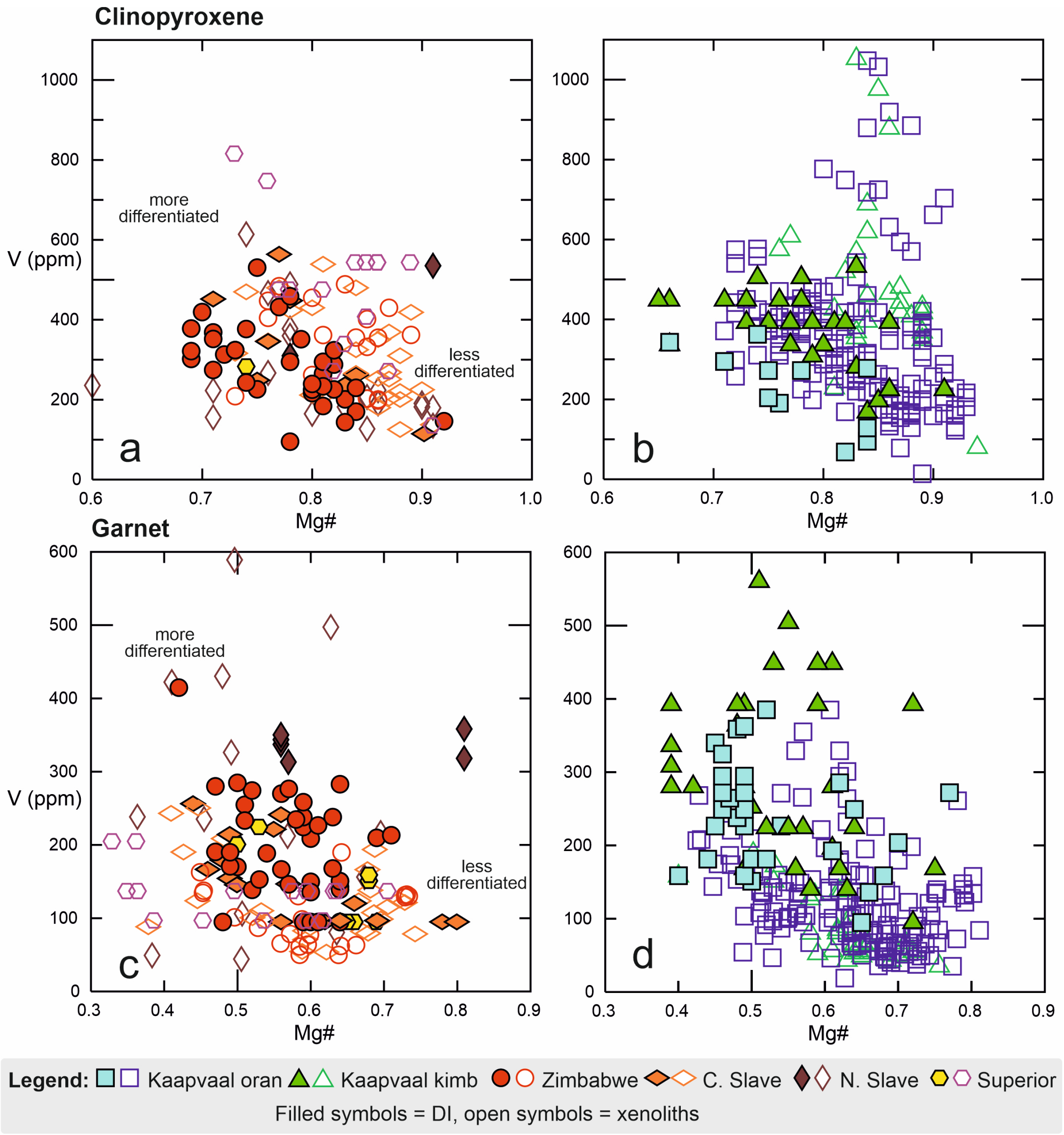

Supplementary Fig. 3 Vanadium abundances (ppm) as a function of Mg# (Mg/(Mg+Fe^{total})), as a proxy for the degree of differentiation during protolith formation. **a-b** Clinopyroxene and **c-d** garnet from eclogite xenoliths and DI. For typical 1σ uncertainties see caption to Supplementary Figure 2.

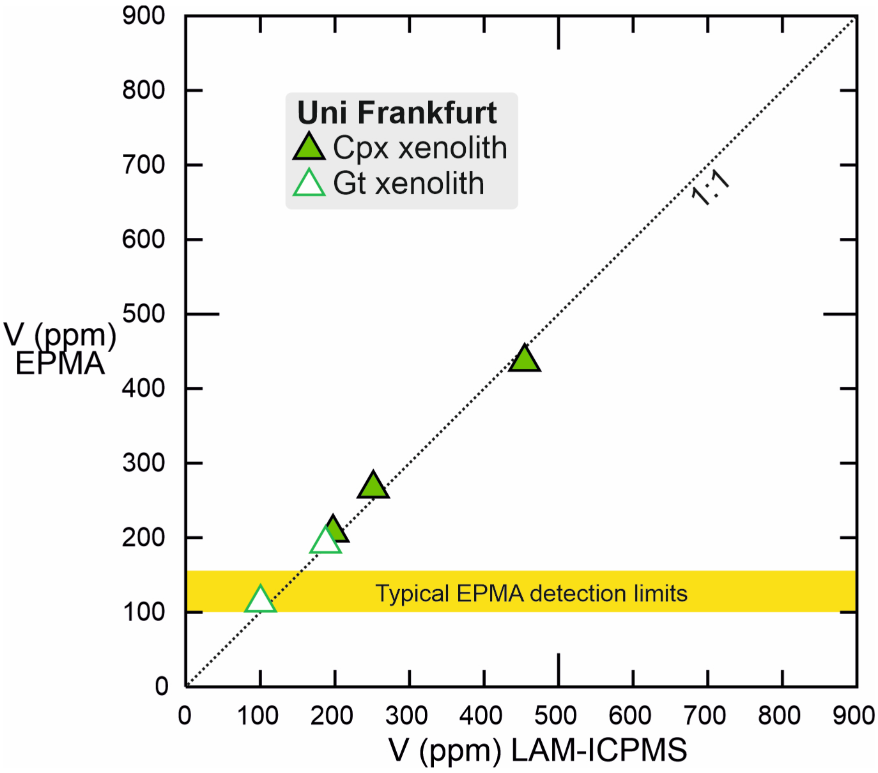

Supplementary Fig. 4 Vanadium abundances (ppm) in clinopyroxene (cpx) and garnet (gt) determined by electron probe microanalyser (EPMA) vs. laser ablation inductively-coupled plasma mass spectrometry (LAM-ICPMS) at Goethe-University Frankfurt. Typical reported detection limits for EPMA are shown as yellow bar.

References for Supplementary Information (including Supplementary Data 1)

1. Jacob, D. E., Viljoen, K. S. & Grassineau, N. V. Eclogite xenoliths from Kimberley, South Africa - A case study of mantle metasomatism in eclogites. *Lithos* **112**, 1002-1013, doi:10.1016/j.lithos.2009.03.034 (2009).
2. Shu, Q., Brey, G. P. & Pearson, D. G. Eclogites and garnet pyroxenites from Kimberley, Kaapvaal craton, South Africa: their diverse origins and complex metasomatic signatures. *Mineralogy and Petrology* **112**, 43-56, doi:10.1007/s00710-018-0595-6 (2018).
3. Smart, K. A. *et al.* Metasomatized eclogite xenoliths from the central Kaapvaal craton as probes of a seismic mid-lithospheric discontinuity. *Chemical Geology* **578**, 120286, doi:https://doi.org/10.1016/j.chemgeo.2021.120286 (2021).
4. Aulbach, S., Viljoen, K. S. & Gerdes, A. Diamondiferous and barren eclogites and pyroxenites from the western Kaapvaal craton record subduction processes and mantle metasomatism, respectively. *Lithos* **368**, doi:10.1016/j.lithos.2020.105588 (2020).
5. Aulbach, S. & Viljoen, K. S. Eclogite xenoliths from the Lace kimberlite, Kaapvaal craton: From convecting mantle source to palaeo-ocean floor and back. *Earth and Planetary Science Letters* **431**, 274-286, doi:10.1016/j.epsl.2015.08.039 (2015).
6. Aulbach, S., Gerdes, A. & Viljoen, K. S. Formation of diamondiferous kyanite-eclogite in a subduction melange. *Geochimica Et Cosmochimica Acta* **179**, 156-176, doi:10.1016/j.gca.2016.01.038 (2016).
7. Burness, S. *et al.* Sulphur-rich mantle metasomatism of Kaapvaal craton eclogites and its role in redox-controlled platinum group element mobility. *Chemical Geology* **542**, 119476, doi:https://doi.org/10.1016/j.chemgeo.2020.119476 (2020).
8. Huang, J.-X., Gréau, Y., Griffin, W. L., O'Reilly, S. Y. & Pearson, N. J. Multi-stage origin of Roberts Victor eclogites: Progressive metasomatism and its isotopic effects. *Lithos* **142**, 161-181, doi:10.1016/j.lithos.2012.03.002 (2012).
9. Schulze, D. J., Valley, J. W. & Spicuzza, M. J. Coesite eclogites from the Roberts Victor Kimberlite, South Africa. *Lithos* **54**, 23-32 (2000).
10. Jacob, D. E., Schmickler, B. & Schulze, D. J. Trace element geochemistry of coesite-bearing eclogites from the Roberts Victor kimberlite, Kaapvaal craton. *Lithos* **71**, 337-351, doi:10.1016/s00244937(03)00120-8 (2003).
11. Radu, I. B., Harris, C., Moine, B. N., Costin, G. & Cottin, J. Y. Subduction relics in the subcontinental lithospheric mantle evidence from variation in the O-18 value of eclogite xenoliths from the Kaapvaal craton. *Contributions to Mineralogy and Petrology* **174**, doi:10.1007/s00410-019-1552-z (2019).
12. Smart, K. A. *et al.* Constraints on Archean crust recycling and the origin of mantle redox variability from the δ44/40Ca – δ18O – fO2 signatures of cratonic eclogites. *Earth and Planetary Science Letters* **556**, 116720 (2021b).
13. Aulbach, S. *et al.* Eclogite xenoliths from Orapa: Ocean crust recycling, mantle metasomatism and carbon cycling at the western Zimbabwe craton margin. *Geochimica Et Cosmochimica Acta* **213**, 574-592, doi:10.1016/j.gca.2017.06.038 (2017).
14. Aulbach, S. *et al.* Ultramafic Carbonated Melt- and Auto-Metasomatism in Mantle Eclogites: Compositional Effects and Geophysical Consequences. *Geochemistry Geophysics Geosystems* **21**, e2019GC008774, doi:10.1029/2019gc008774 (2020).
15. Smart, K. A., Chacko, T., Simonetti, A., Sharp, Z. D. & Heaman, L. M. A Record of Paleoproterozoic Subduction Preserved in the Northern Slave Cratonic Mantle: Sr-Pb-O Isotope and Trace-element Investigations of Eclogite Xenoliths from the Jericho and Muskox Kimberlites. *Journal of Petrology* **55**, 549-583, doi:10.1093/petrology/egt077 (2014).
16. Smart, K. A. *et al.* Tectonic significance and redox state of Paleoproterozoic eclogite and pyroxenite components in the Slave cratonic mantle lithosphere, Voyageur kimberlite, Arctic Canada. *Chemical Geology* **455**, 98-119, doi:10.1016/j.chemgeo.2016.10.014 (2017).
17. Aulbach, S., Pearson, N. J., O'Reilly, S. Y. & Doyle, B. J. Origins of xenolithic eclogites and pyroxenites from the central slave craton, Canada. *Journal of Petrology* **48**, 1843-1873 (2007).
18. Schmidberger, S. S., Simonetti, A., Heaman, L. M., Creaser, R. A. & Whiteford, S. Lu-Hf, in-situ Sr and Pb isotope and trace element systematics for mantle eclogites from the Diavik diamond mine: Evidence for Paleoproterozoic subduction beneath the Slave craton, Canada.

Earth and Planetary Science Letters **254**, 55-68, doi:10.1016/j.epsl.2006.11.020 (2007).

19 Aulbach, S., Stachel, T., Heaman, L. M. & Carlson, J. A. Microxenoliths from the Slave craton: Archives of diamond formation along fluid conduits. *Lithos* **126**, 419-434, doi:10.1016/j.lithos.2011.07.012 (2011).

20 Smit, K. V. *et al.* Origin of eclogite and pyroxenite xenoliths from the Victor kimberlite, Canada, and implications for Superior craton formation. *Geochimica Et Cosmochimica Acta* **125**, 308-337, doi:10.1016/j.gca.2013.10.019 (2014).

21 Phillips, D., Harris, J. W. & Viljoen, K. S. Mineral chemistry and thermobarometry of inclusions from De Beers Pool diamonds, Kimberley, South Africa. Lithos 77, 155-179 (2004).

22 Stachel, T. Stachel, Thomas, 2021, Diamond Inclusion Database, Scholars Portal Dataverse, V1 (2021). https://dataverse.scholarsportal.info/dataset.xhtml?persistentId=doi:10.7939/DVN/EJUE1G

23 Viljoen, K. S., Perritt, S. H. & Chinn, I. L. An unusual suite of eclogitic, websteritic and transitional websteritic-lherzolitic diamonds from the Voorspoed kimberlite in South Africa: Mineral inclusions and infrared characteristics. *Lithos* **320**, 416-434, doi:10.1016/j.lithos.2018.09.034 (2018).

24 Deines, P., Stachel, T. & Harris, J. W. Systematic regional variations in diamond carbon isotopic composition and inclusion chemistry beneath the Orapa kimberlite cluster, in Botswana. *Lithos* **112**, 776-784, doi:10.1016/j.lithos.2009.03.027 (2009).

25 Motsamai, T., Harris, J. W., Stachel, T., Pearson, D. G. & Armstrong, J. Mineral inclusions in diamonds from Karowe Mine, Botswana: super-deep sources for super-sized diamonds? *Mineralogy and Petrology* **112**, 169-180, doi:10.1007/s00710-018-0604-9 (2018).

26 De Stefano, A., Kopylova, M. G., Cartigny, P. & Afanasiev, V. Diamonds and eclogites of the Jericho kimberlite (Northern Canada). *Contributions to Mineralogy and Petrology* **158**, 295-315, doi:10.1007/s00410-009-0384-7 (2009).

27 Davies, R. A., Griffin, W. L., O'Reilly, S. Y. & Doyle, B. J. Mineral inclusions and geochemical characteristics of microdiamonds from the DO27, A154, A21, A418, DO18, DD17 and Ranch Lake kimberlites at Lac de Gras, Slave Craton, Canada. *Lithos* **77**, 39-55, doi:10.1016/j.lithos.2004.04.016 (2004).

28 Donnelly, C. L., Stachel, T., Creighton, S., Muehlenbachs, K. & Whiteford, S. Diamonds and their mineral inclusions from the A154 South pipe, Diavik Diamond Mine, Northwest Territories, Canada. *Lithos* **98**, 160-176, doi:10.1016/j.lithos.2007.03.003 (2007).

29 Promprated, P. *et al.* Multiple-mineral inclusions in diamonds from the Snap Lake/King Lake kimberlite dike, Slave craton, Canada: a trace-element perspective. *Lithos* **77**, 69-81, doi:https://doi.org/10.1016/j.lithos.2004.04.009 (2004).

30 Stachel, T. *et al.* The Victor Mine (Superior Craton, Canada): Neoproterozoic lherzolitic diamonds from a thermally-modified cratonic root. *Mineralogy and Petrology* **112**, 325-336, doi:10.1007/s00710-018-0574-y (2018).

31 Gale, A., Laubier, M., Escrig, S. & Langmuir, C. H. Constraints on melting processes and plume-ridge interaction from comprehensive study of the FAMOUS and North Famous segments, Mid-Atlantic Ridge. *Earth and Planetary Science Letters* **365**, 209-220, doi:10.1016/j.epsl.2013.01.022 (2013).

32 Donovan, J. J., Lowers, H. A. & Rusk, B. G. Improved electron probe microanalysis of trace elements in quartz. *American Mineralogist* **96**, 274-282, doi:doi:10.2138/am.2011.3631 (2011).

33 Wang, J. T. *et al.* Oxidation State of Arc Mantle Revealed by Partitioning of V, Sc, and Ti Between Mantle Minerals and Basaltic Melts. *Journal of Geophysical Research-Solid Earth* **124**, 4617-4638, doi:10.1029/2018jb016731 (2019).

34 Katsura, T., Yoneda, A., Yamazaki, D., Yoshino, T. & Ito, E. Adiabatic temperature profile in the mantle. *Physics of the Earth and Planetary Interiors* **183**, 212-218, doi:10.1016/j.pepi.2010.07.001 (2010).

35 Herzberg, C. & Asimow, P. D. Petrology of some oceanic island basalts: PRIMELT2.XLS software for primary magma calculation. *Geochemistry Geophysics Geosystems* **9**, doi:10.1029/2008gc002057 (2008).

36 Aulbach, S. & Jacob, D. E. Major- and trace-elements in cratonic mantle eclogites and pyroxenites reveal heterogeneous sources and metamorphic processing of low-pressure protoliths. *Lithos* **262**, 586-605, doi:10.1016/j.lithos.2016.07.026 (2016).

37 Holycross, M. & Cottrell, E. Partitioning of V and 19 other trace elements between rutile and

silicate melt as a function of oxygen fugacity and melt composition: Implications for subduction zones. *American Mineralogist* **105**, 244-254, doi:10.2138/am-2020-7013 (2020).
38 Mallmann, G. & O'Neill, H. S. C. The Crystal/Melt Partitioning of V during Mantle Melting as a Function of Oxygen Fugacity Compared with some other Elements (Al, P, Ca, Sc, Ti, Cr, Fe, Ga, Y, Zr and Nb). *Journal of Petrology* **50**, 1765-1794, doi:10.1093/petrology/egp053 (2009).
39 Barth, M. G., Foley, S. F. & Horn, I. Partial melting in Archean subduction zones: constraints from experimentally determined trace element partition coefficients between eclogitic minerals and tonalitic melts under upper mantle conditions. *Precambrian Research* **113**, 323-340 (2002).
40 Tappe, S. *et al.* Plates or plumes in the origin of kimberlites: U/Pb perovskite and Sr-Nd-Hf-Os-C-O isotope constraints from the Superior craton (Canada). *Chemical Geology* **455**, 57-83, doi:10.1016/j.chemgeo.2016.08.019 (2017).
41 Krogh, E. J. The garnet-clinopyroxene Fe-Mg geothermometer - a reinterpretation of existing experimental data. *Contributions to Mineralogy and Petrology* **99**, 44-48, doi:10.1007/bf00399364 (1988).
42 Jenner, F. E. & O'Neill, H. S. Analysis of 60 elements in 616 ocean floor basaltic glasses. *Geochemistry Geophysics Geosystems* **13**, Q02005, doi:10.1029/2011gc004009 (2012).
