# Supplementary material for: Evidence for oxygen-conserving diamond formation in redox-buffered subducted oceanic crust sampled as eclogite
Source: Nat Commun. 2022 Apr 8;13:1924. doi: 10.1038/s41467-022-29567-z (PMC8993838; doi:10.1038/s41467-022-29567-z)
Supplement: Supplementary file 3 — Description of Additional Supplementary Files [file 41467_2022_29567_MOESM3_ESM.docx]

Description of Additional Supplementary Files

File name: Supplementary Data 1

Description: Salient clinopyroxene, garnet and reconstructed whole-rock and rutile compositions, and temperature-ƒO2 estimates from the literature, as well as statistical evaluation of data for xenoliths vs. inclusions in diamond.
