# Supplementary material for: Author Correction: Evidence for oxygen-conserving diamond formation in redox-buffered subducted oceanic crust sampled as eclogite
Source: Nat Commun. 2026 Feb 2;17:1209. doi: 10.1038/s41467-026-69139-z (PMC12864721; doi:10.1038/s41467-026-69139-z)
Supplement: Supplementary file 1 — Explanation of changes [file 41467_2026_69139_MOESM1_ESM.pdf]

Corrections to the main text are detailed below, with the changes shown in red.

(1) Text section "RESULTS, Temperature and crystal-chemical effects on V distribution":

Original:
However, a notable difference between garnet in eclogitic DI and xenoliths is the higher equilibration temperatures recorded by the former at the time of encapsulation in diamond (median of 1010 °C for DI vs. 890 °C for xenoliths) (Fig. 1c) under generally warmer mantle conditions in Archaean and Palaeoproterozoic time. Indeed, in four of six sample suites...

Corrected
However, a notable difference between garnet in eclogitic DI and xenoliths is the higher equilibration temperatures recorded by the former at the time of encapsulation in diamond (median of 1154 °C for DI vs. 1096 °C for xenoliths) (Fig. 1c) under generally warmer mantle conditions in Archaean and Palaeoproterozoic time. Indeed, in three of six sample suites…

Original
Median TiO_2 contents in DI garnet (0.56 wt.%) are higher than those in xenolith garnet (0.18 wt.%) (Fig. 1e), and TiO_2 in garnet is significantly positively correlated with temperature in four of six sample suites (Table 2)...

Corrected
Median TiO_2 contents in DI garnet (0.56 wt.%) are higher than those in xenolith garnet (0.18 wt.%) (Fig. 1e), and TiO_2 in garnet is significantly positively correlated with temperature in all six sample suites (Table 2)…

(2) Text section "RESULTS, Oxygen fugacity effects on V partitioning in eclogitic minerals"

Original
$D(V)_{cpx-gt}$ in eclogite xenoliths shows no significant correlation with fO_2 when all data are considered (Table 2), but a weak one ($r^2 = 0.26$, $n = 15$) for samples from the Zimbabwe and the central Slave cratons. While V abundances in clinopyroxene show a very weak dependence on fO_2 ($r^2 = 0.12$; $n = 59$; Fig. 2b; Table 2), those in garnet show a stronger positive correlation ($r^2 = 0.23$; $n = 59$; Fig. 2c; Table 2).

Corrected
$D(V)_{cpx-gt}$ in eclogite xenoliths shows a significant correlation with fO_2 when all data are considered (Table 2). While V abundances in clinopyroxene show no dependence on fO_2 (r^2= 0.06; n = 59; Fig. 2b; Table 2), those in garnet show a stronger positive correlation (r^2= 0.23; n = 59; Fig. 2c; Table 2).

(3) Text section "DISCUSSION"

Original
In contrast, this study makes the intriguing observation that with increasing fO_2, V abundances in eclogite minerals (Fig. 2b, c) and bulk rocks (Fig. 4b) from some suites show a mild but statistically significant increase (Table 2) or remain constant for others.

Corrected

In contrast, this study makes the intriguing observation that with increasing fO_2, V abundances in eclogite garnet (Fig. 2b-c) and bulk rocks (Fig. 4b) show a mild but statistically significant increase (Table 2).

The original Tables 1 and 2 and Figs. 1, 2 and 4 are shown below.

Table 1 Salient parameters of clinopyroxene, garnet and reconstructed whole rocks in eclogite xenoliths and inclusions in diamond (DI), as well as temperature-fO_2 estimates from the literature (references in Supplementary Data 1).

	Clinopyroxene					Cpx/Gt
	TiO_2	Al_2O_3/FeO^t	Mg#	Jd	V	*D*(V)
Unit	wt.%				ppm	
Xenoliths *n* =	261	261	261	261	261	261
Min	0.04	0.4	0.6	0.13	14	0.16
Max	0.83	15.5	0.94	0.58	1050	10.9
Mean	0.27	2.9	0.83	0.31	360	3.4
1σ	0.11	2.8	0.06	0.11	178	1.83
Median	0.26	1.87	0.84	0.31	350	3
DI *n* =	85	85	85	85	87	17
Min	0.04	0.21	0.65	0.05	68	0.86
Max	0.84	14.7	0.92	0.45	560	2.8
Mean	0.43	1.57	0.78	0.26	320	1.47
1σ	0.2	2.1	0.06	0.11	114	0.63
Median	0.41	1.17	0.78	0.27	320	1.2
Means different?[a]	Yes	Yes	Yes	No	Yes	Yes

	Garnet					
	TiO_2	Al_2O_3/FeO^t	Na_2O	Mg#	Ca#	V
Unit	wt.%	wt.%	wt.%			ppm
Xenoliths *n* =	258	259	255	259	259	261
Min	0.02	0.83	0.01	0.25	0.06	19
Max	0.99	3.4	0.24	0.81	0.51	920
Mean	0.22	1.6	0.08	0.61	0.2	141
1σ	0.14	0.45	0.04	0.1	0.09	123
Median	0.18	1.49	0.07	0.63	0.19	110
DI *n* =	135	135	135	135	135	134
Min	0.12	0.91	0.03	0.39	0.05	95
Max	1.7	2.7	0.54	0.81	0.58	560
Mean	0.63	1.45	0.2	0.57	0.24	230
1σ	0.30	0.35	0.12	0.09	0.11	99
Median	0.56	1.38	0.17	0.56	0.23	220
Means different?[a]	Yes	Yes	Yes	Yes	Yes	Yes

	Reconstructed whole rocks[b]				Oxythermobarometry[c]	
	Al_2O_3/FeO^t	MgO	V	Eu/Eu*	Log fO_2	*T*
Unit	wt.% basis	wt.%	ppm		ΔFMQ	°C
Xenoliths *n* =	258	258	260	258	59	259
Min	0.84	5.5	35	0.69	−5.6	630
Max	4.3	18.6	670	2.70	−1.3	1440
Mean	1.74	12.4	250	1.14	−3.3	890
1σ	0.6	2.6	115	0.29	0.96	126
Median	1.57	12.2	230	1.07	−3.3	890
DI *n* =	18	18	17	5		18
Min	0.87	8.1	95	0.94		830
Max	2.5	16	480	1.15		1270
Mean	1.32	12	270	1.03		1020
1σ	0.38	2.2	105	0.09		136
Median	1.32	11.9	250	0.99		1010
Means different?[a]	Yes	No	No	Yes	Na	Yes

Mg# Mg/(Mg + Fe^{total}), *Jd* jadeite component in clinopyroxene (cpx), *Ca#* Ca/(Ca + Mg + Fe^{total} + Mn) in garnet (gt), *D* distribution coefficient, *Eu/Eu** chondrite-normalised Eu/$(Sm*Gd)^{0.5}$ (chondrite of ref. [69]).

[a]Based on two-tailed *t*-test for null hypothesis that the means of two populations are equal, and imposing alpha = 0.05 (below which the null hypothesis is rejected), using either equal or unequal variances depending on F-test outcomes

[b]Bulk rocks are reconstructed with 0.55 garnet, 0.45 clinopyroxene minus half the weight of rutile each (e.g., for 1 wt.% rutile 0.545 garnet, 0.445 clinopyroxene); for V reconstruction, estimated rutile modes and concentrations in rutile were considered.

[c]Oxygen fugacities as reported or recalculated relative to the Fayalite-Magnetite-Quartz (FMQ) buffer using the eclogite oxybarometer of ref. [70]; temperatures are derived using the thermometer of ref. [51] in iterative solution with regional conductive model geotherms (see ref. [21]).

Table 2 Pearson correlation coefficents and statistical significance test for various variables discussed in the text (references in Supplementary Data 1).

Suite	$D(V)_{cpx\text{-}gt}$-T (°C)			Gt TiO_2 (wt.%)-T (°C)			Gt V (ppm)-Ca#[a]			Gt Na_2O (wt.%)-T (°C)		
	**r**	***n***	***p***	**r**	***n***	***p***	**r**	***n***	***p***	**r**	***n***	***p***
1	0.6	36	<0.001	0.22	36	0.196	0.11	34	0.543	0.63	32	<0.001
2	0.22	154	0.006	0.23	154	0.005	0.15	151	0.067	0.32	154	<0.001
3	0.86	27	<0.001	0.5	27	0.007	0.92	21	<0.001	0.83	27	<0.001
4	0.16	20	0.488	0.66	21	0.001	0.46	20	0.041	0.84	21	<0.001
5	0.77	24	<0.001	0.85	15	<0.001	0.38	24	0.067	0.55	24	0.006
6	0.1	15	0.718	0.28	24	0.186	0.19	15	0.488	0.3	15	0.271

	Gt V (ppm)-TiO_2 (wt.%)			Gt V (ppm)-Na_2O (wt.%)			Gt Na_2O-TiO_2 (wt.%)		
	**r**	***n***	***p***	**r**	***n***	***p***	**r**	***n***	***p***
1	0.88	61	<0.001	0.42	57	0.001	0.66	61	<0.001
2	0.53	181	<0.001	0.45	182	<0.001	0.93	181	<0.001
3	0.67	61	<0.001	0.57	61	<0.001	0.86	61	<0.001
4	0.18	26	0.385	0.32	26	0.106	0.76	27	<0.001
5	0.48	41	0.001	0.27	41	0.083	0.74	41	<0.001
6	0.41	22	0.059	0.47	22	0.027	0.8	22	<0.001

	$D(V)_{cpx\text{-}gt}$-fO_2			Cpx V (ppm)-fO_2			Gt V (ppm)-fO_2			WR V (ppm)-fO_2		
	**r**	***n***	***p***	**r**	***n***	***p***	**r**	***n***	***p***	**r**	***n***	***p***
All	0.23	59	0.083	0.34	59	0.008	0.48	59	<0.001	0.41	59	0.001

Suites: 1—Kaapvaal craton kimberlite-hosted, 2—Kaapvaal craton orangeite-hosted, 3—Zimbabwe craton, 4—Northern Slave craton, 5—Central Slave craton, 6—Superior craton.
r—Pearson correlation coefficient, n—number of observations, *p*—*p*-value for statistical signficance testing, whereby a value of ≤0.05 is taken to indicate statistical significance (i.e., the null hypothesis of no significant correlation is rejected).
Cpx clinopyroxene, *Gt* garnet, *Ca#* molar Ca/(Ca + Mg + Fe^{total} + Mn), *D* distribution coefficient, oxygen fugacity values fO_2 and temperatures as in Table 1 .
[a]Xenoliths only to minimise superposition of temperature effects.

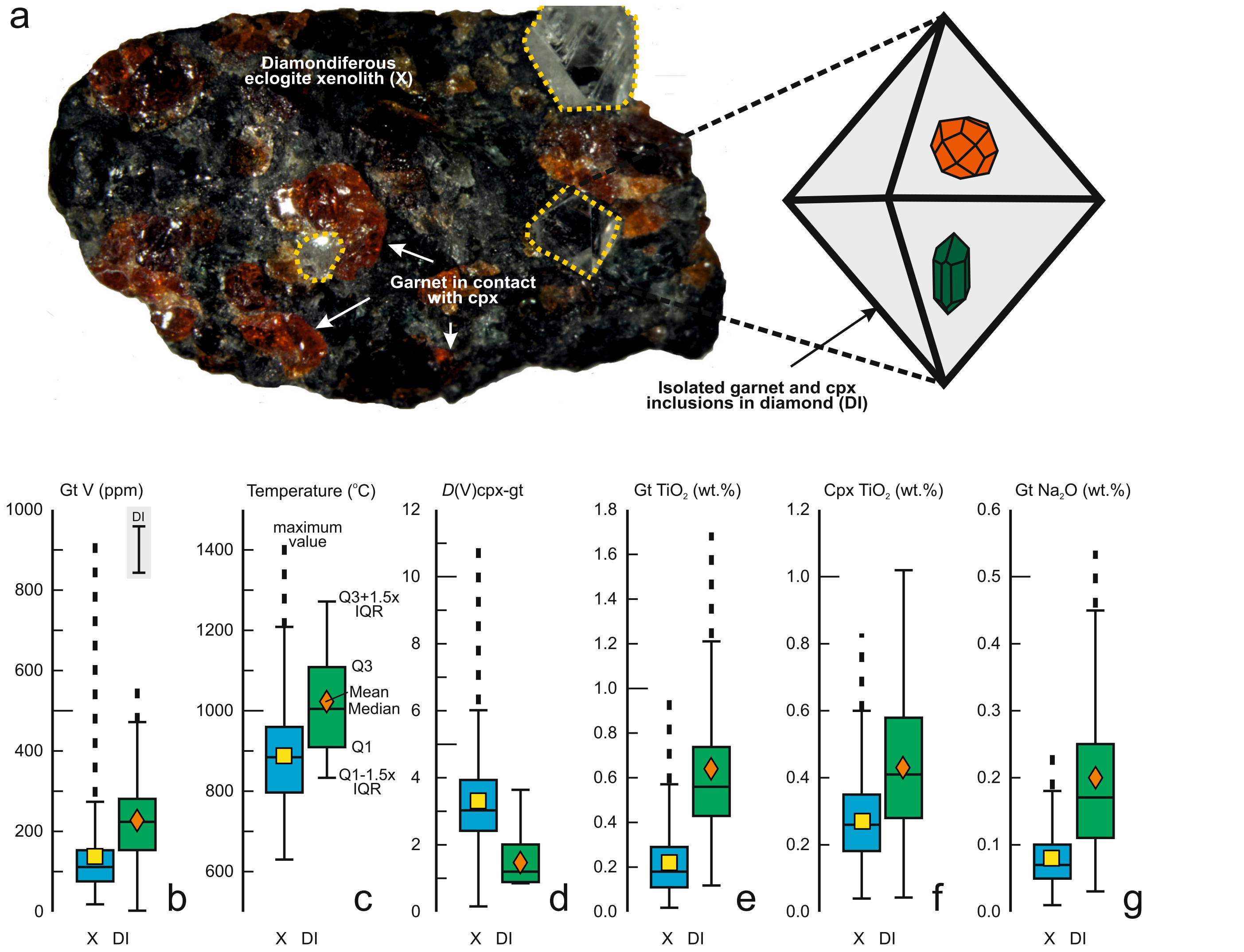

Fig. 1 Illustration of the closed-system behaviour of isolated eclogitic inclusions in diamond (DI) vs. open-system behaviour of touching inclusions in eclogite xenoliths (X). a Photograph of a diamondiferous eclogite xenolith from Fort à la Corne (~1.5 cm longest dimension); diamond crystals are highlighted in yellow stipples. Also shown is the schematic of a diamond hosting eclogitic garnet and clinopyroxene inclusions that are non-touching, which prevents them from chemically re-equilibrating to lower temperatures, while the inert diamond also protects them from communicating with the open-system matrix. Contrasting temperature estimates and compositions shown as box-and-whisker plots: **b** Vanadium concentrations (ppm) in garnet (gt), **c** temperature estimates (°C) reflecting those of the eclogite sources at the time of xenolith entrainment in kimberlite, vs. the time of encapsulation of non-touching inclusions in diamond, **d** Distribution (*D*) of V between clinopyroxene (cpx) and garnet ($D(V)_{cpx\text{-}gt}$), **e** TiO_2 content (wt.%) in garnet, **f** TiO_2 content (wt.%) in clinopyroxene, **g** Na_2O content (wt.%) in garnet. Explanations for the statistical parameters displayed in this figure (mean, median, Q1 first quartile, Q3 third quartile) are shown in panel **c**. The whiskers are here defined as Q3 + 1.5×IQR (interquartile range), and Q1-1.5×IQR or the minimum value, whichever is higher; stippled lines extend to the maximum value if >Q3 + 1.5×IQR. Average 1σ uncertainty on V abundances in garnet from DI of 117 ppm is shown as error bar; average 1σ uncertainty on V abundances in garnet from xenoliths of 5.6 ppm is not displayed at the scale; both correspond to typical uncertainties for multiple analyses per sample reported in the literature, see Methods). Data sources in Supplementary Data 1.

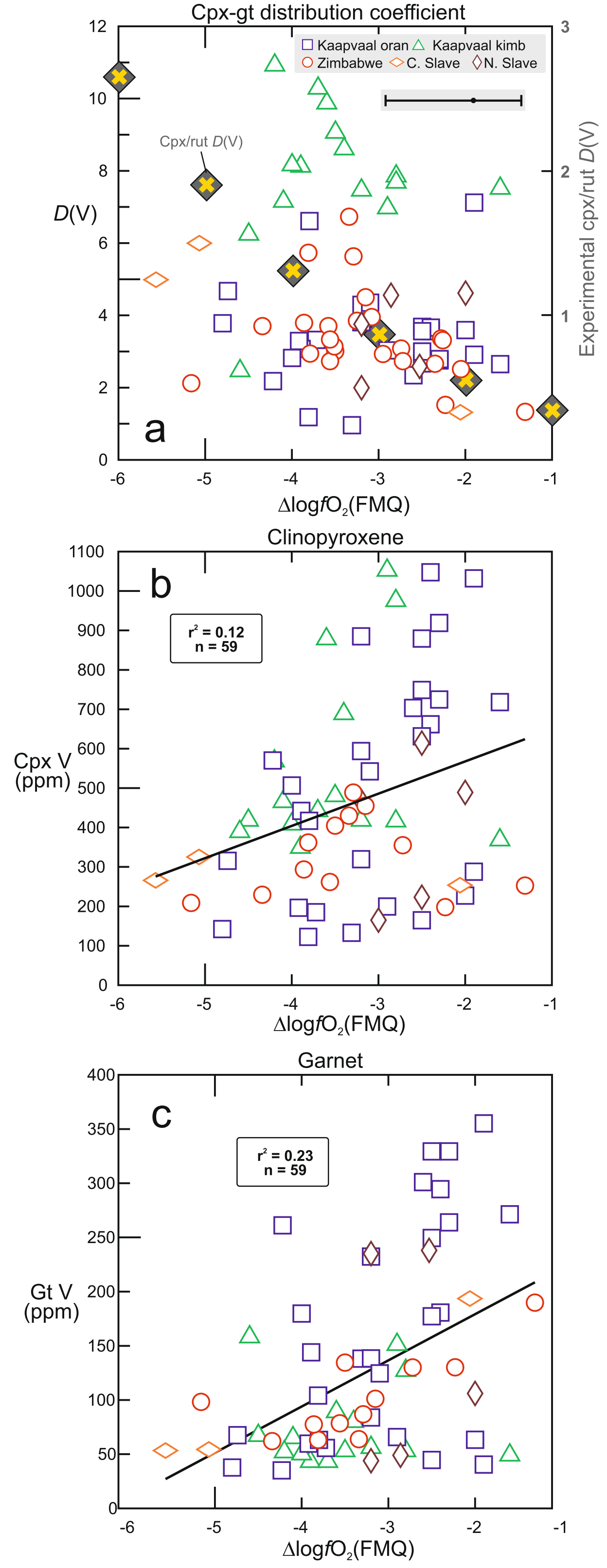

Fig. 2 Effects of oxygen fugacity on the distribution and abundances of V in eclogite minerals. a Distribution *D* of V between clinopyroxene (cpx) and garnet (gt) in eclogite xenoliths as a function of oxygen fugacity relative to the Fayalite-Magnetite-Quartz buffer ($\Delta \log fO_2$(FMQ); available for xenoliths only); oran-orangeite-hosted, kimb-kimberlite-hosted. Superposed is the distribution of V between clinopyroxene and rutile (rut) as a function of fO_2 (yellow crosses), as calculated from the parameterisation of experimentally determined mineral-melt partition coefficients[12,26]. Vanadium concentration (ppm) in **b** clinopyroxene and **c** garnet as a function of fO_2, with regressions and r^2 (significant for the number n of observations; Table 2). Error bar in **a** shows representative average 1σ uncertainty of fO_2 estimates of −1.0, +0.6[14]; 1σ uncertainties on V abundances of 14.5 ppm for clinopyroxene and 5.6 ppm for garnet (corresponding to typical uncertainties for multiple analyses per sample reported in the literature, see Methods) are small relative to the depicted scale and not shown. Data sources in Supplementary Data 1.

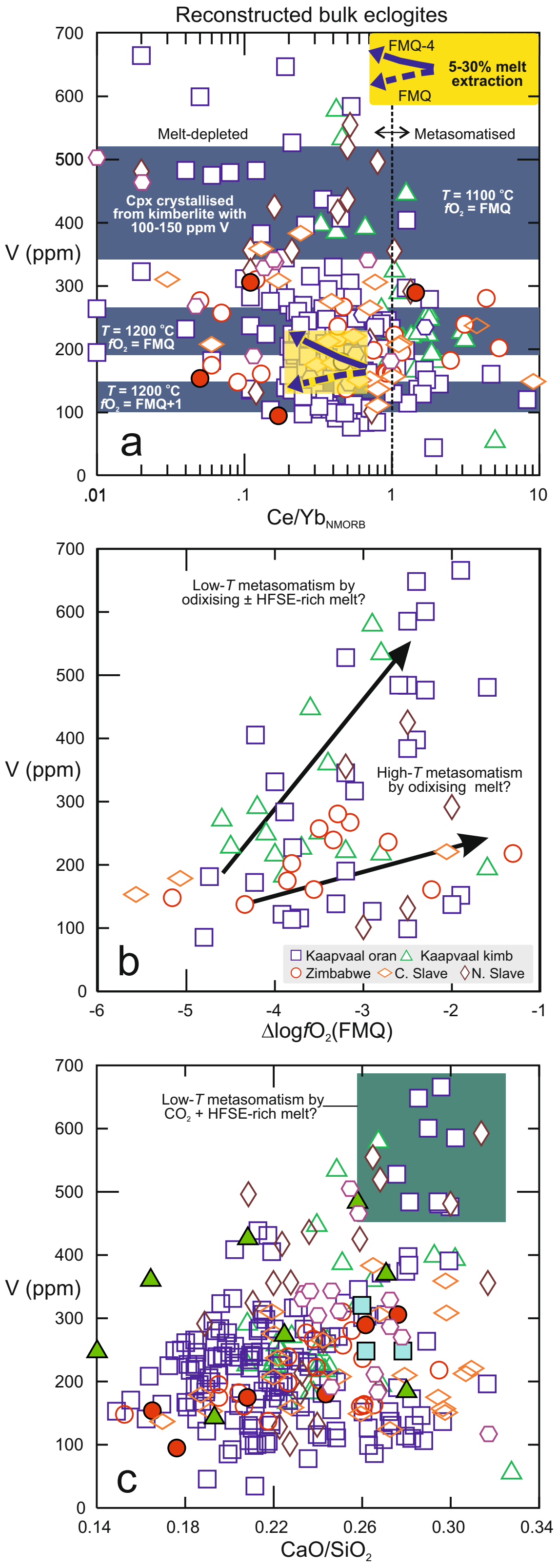

Fig. 4 Effects of partial melt extraction and metasomatism on V abundances in eclogites. a Vanadium abundances (ppm) in reconstructed bulk eclogite xenoliths and DI as a function of normal mid-ocean ridge basalt (NMORB)-normalised Ce/Yb, a proxy for melt depletion and enrichment. Shown for comparison is the modelled trend (yellow box) for partial melt extraction from rutile-eclogite, with a moderate decrease of V abundances at relatively oxidising conditions of $\Delta \log fO_2(FMQ) = 0$, similar to the modern ambient mantle[72]. For $\Delta \log fO_2(FMQ) = -4$, at the low end of fO_2 estimates for eclogite xenoliths, partial melt extraction from eclogite causes an increase in V abundances (modelling parameters and rationale in Methods; Supplementary Table 4). Also shown is the effect of metasomatism, as mediated by crystallisation of a high-temperature pyroxene from a kimberlite melt[21] with 100–150 ppm V[66]; V abundances in the metasomatic clinopyroxene are calculated for various temperature and fO_2 conditions, shown with grey-blue fields (modelling parameters and rationale in Methods; Supplementary Table 5); reconstructed pre-metasomatic eclogites ($Cr_2O_3 < 0.1$ and $Ce/Yb_{NMORB} < 1$) would have 280 ± 140 ppm V. NMORB for normalisation from ref. [60]. **b** V abundances (ppm) in reconstructed bulk eclogite xenoliths as a function of oxygen fugacity relative to the Fayalite-Magnetite-Quartz buffer ($\Delta \log fO_2(FMQ)$; no combined fO_2-V information available for DI), showing an overall positive correlation (significant, see Table 2). Arrows qualitatively indicate effects of melt metasomatism via clinopyroxene addition under various conditions as shown in **a** (HFSE-high field-strength elements). **c** V abundances (ppm) in reconstructed bulk eclogite xenoliths and DI as a function of CaO/SiO_2, a proxy for metasomatism by CO_2-rich melt[49]; note samples with anomalous V enrichment are restricted to high ratios (teal-coloured field). Average propagated 1σ uncertainties on V abundances are 29 ppm for xenoliths and 69 ppm for DI (Methods). Data sources in Supplementary Data 1.
